# Protective mechanism of artemisinin on rat bone marrow-derived mesenchymal stem cells against apoptosis induced by hydrogen peroxide via activation of c-Raf-Erk1/2-p90^rsk^-CREB pathway

**DOI:** 10.1186/s13287-019-1419-2

**Published:** 2019-10-26

**Authors:** Jiankang Fang, Xia Zhao, Shuai Li, Xingan Xing, Haitao Wang, Philip Lazarovici, Wenhua Zheng

**Affiliations:** 1 Centre of Reproduction, Development and Aging, Institute of Translational Medicine, Faculty of Health Sciences, University of Macau, Macau SAR, China; 2 School of Pharmaceutical Sciences, Sothern Medical University, Guangzhou, China; 3 School of Pharmacy Institute for Drug Research, Faculty of Medicine, The Hebrew University of Jerusalem, 91120 Jerusalem, Israel

**Keywords:** Artemisinin, Bone marrow mesenchymal stem cells, Apoptosis, ROS, Erk1/2, Raf, p90^rsk^, CREB, Phosphorylation, Protection

## Abstract

**Background:**

Bone marrow-derived mesenchymal stem cell (BMSC) transplantation is one of the new therapeutic strategies for treating ischemic brain and heart tissues. However, the poor survival rate of transplanted BMSCs in ischemic tissue, due to high levels of reactive oxygen species (ROS), limits the therapeutic efficacy of this approach. Considering that BMSC survival may greatly enhance the effectiveness of transplantation therapy, development of effective therapeutics capable of mitigating oxidative stress-induced BMSC apoptosis is an important unmet clinical need.

**Methods:**

BMSCs were isolated from the 4-week-old male Sprague Dawley rats by whole bone marrow adherent culturing, and the characteristics were verified by morphology, immunophenotype, adipogenic, and osteogenic differentiation potential. BMSCs were pretreated with artemisinin, and H_2_O_2_ was used to induce apoptosis. Cell viability was detected by MTT, FACS, LDH, and Hoechst 33342 staining assays. Mitochondrial membrane potential (ΔΨm) was measured by JC-1 assay. The apoptosis was analyzed by Annexin V-FITC/PI and Caspase 3 Activity Assay kits. ROS level was evaluated by using CellROX® Deep Red Reagent. SOD, CAT, and GPx enzymatic activities were assessed separately using Cu/Zn-SOD and Mn-SOD Assay Kit with WST-8, Catalase Assay Kit, and Total Glutathione Peroxidase Assay Kit. The effects of artemisinin on protein expression of BMSCs including p-Erk1/2, t-Erk1/2, p-c-Raf, p-p90^rsk^, p-CREB, BCL-2, Bax, p-Akt, t-Akt, β-actin, and GAPDH were measured by western blotting.

**Results:**

We characterized for the first time the protective effect of artemisinin, an anti-malaria drug, using oxidative stress-induced apoptosis in vitro, in rat BMSC cultures. We found that artemisinin, at clinically relevant concentrations, improved BMSC survival by reduction of ROS production, increase of antioxidant enzyme activities including SOD, CAT, and GPx, in correlation with decreased Caspase 3 activation, lactate dehydrogenase (LDH) release and apoptosis, all induced by H_2_O_2_. Artemisinin significantly increased extracellular-signal-regulated kinase 1/2 (Erk1/2) phosphorylation, in a concentration- and time-dependent manner. PD98059, the specific inhibitor of the Erk1/2 pathway, blocked Erk1/2 phosphorylation and artemisinin protection. Similarly, decreased expression of Erk1/2 by siRNA attenuated the protective effect of artemisinin. Additionally, when the upstream activator KRAS was knocked down by siRNA, the protective effect of artemisinin was also blocked. These data strongly indicated the involvement of the Erk1/2 pathway. Consistent with this hypothesis, artemisinin increased the phosphorylation of Erk1/2 upstream kinases proto-oncogene c-RAF serine/threonine-protein kinase (c-Raf) and of Erk1/2 downstream targets p90 ribosomal s6 kinase (p90^rsk^) and cAMP response element binding protein (CREB). In addition, we found that the expression of anti-apoptotic protein B cell lymphoma 2 protein (BcL-2) was also upregulated by artemisinin.

**Conclusion:**

These studies demonstrate the proof of concept of artemisinin therapeutic potential to improve survival in vitro of BMSCs exposed to ROS-induced apoptosis and suggest that artemisinin-mediated protection occurs via the activation of c-Raf-Erk1/2-p90^rsk^-CREB signaling pathway.

## Introduction

BMSCs are multipotent stem cells derived from the bone marrow (BM) stem cell niche. In recent years, there has been a huge interest to isolate, culture, and characterize these BMSCs due to their therapeutic potential in regenerative medicine [1]. For purposes of experimental and therapeutic use, freshly obtained BMSCs are cultured in the plastic adherent dishes, thus providing a heterogeneous population of cells which are plastic-adherent in culture and express the typical mesenchymal markers *CD29*, *CD73*, *CD90*, and *CD105* but lack expression of the typical hematopoietic markers *CD11b*, *CD14*, *CD34*, *CD45*, and *CD79α*, and have the capacity to differentiate in vitro into osteoblasts, adipocytes, and chondroblasts [2, 3]. Although mesenchymal stem cells are commonly believed to adjust with oxidative stress [4–6], the biggest obstacle to their therapeutic use is their poor survival in ischemic tissue targets after engraftment [7–9]. Therefore, studies focusing on how to protect transplanted BMSCs against oxidative stress-induced apoptosis become a key issue for the success of BMSC transplantation [10]. Pathological levels of ROS generated at the ischemic site of tissue injury have been hypothesized to lead to loss of transplanted BMSCs from this site [11, 12]. Therefore, there is great need to identify therapies that might manipulate BMSCs to reduce ROS in both the BMSCs themselves during their culture expansion production phase and upon homing to the injured tissue microenvironment, in order to promote BMSC engraftment and enhance tissue repair.

Artemisinin, a sesquiterpene endoperoxide, discovered by the pharmacist Tu Youyou, is a famous natural compound from Chinese medical herb *Artemisia annua.* The related compounds, such as artemisinic acid, dihydroartemisinin, artesunate, artemether, and arteether are all derivatives of artemisinin. During the last several years, artemisinin-based combination treatments (ACTs) became the frontline therapy for uncomplicated malaria caused by *P. falciparum* [13–15]. Moreover, several studies indicated that artemisinin and its derivatives may be beneficial in other clinical applications by conferring different activities such as anti-inflammatory [16–20], anti-viral [21], anti-microbial [22–24], anti-cancer [25–29], immunomodulatory [17, 30], anti-fungal [31], and anti-diabetic [32, 33]. Also, there are in vitro evidences indicating the protective activity of artemisinin towards oxidative stress damage [34–38]. Thus, the main goals of the present study were to examine whether artemisinin confers cytoprotection via its antioxidant properties and to clarify whether the c-Raf/Erk1/2/p90^rsk^/CREB pathway is critical in mediating artemisinin protection towards H_2_O_2_ oxidative stress-induced apoptosis in BMSCs.

The present study demonstrates that artemisinin can protect BMSCs from oxidative stress possibly by activating the c-Raf/Erk1/2/p90^rsk^/CREB pathway.

## Materials and methods

### Materials

MEM medium was purchased from HyClone, Logan, UT, USA; 3-(4, 5-dimethylthiazol-2-yl)-2, 5-diphenyltetrazolium bromide (MTT) and Hoechst 33342 solution were bought from Molecular Probes, Eugene, OR, USA; CellROX® Deep Red Reagent was obtained from Thermo Fisher Scientific, Waltham, MA, USA; Anti-rat CD45 FITC (#11-0461-80) and Anti-mouse/rat CD 90.1(Thy-1.1) FITC (#11-0900-81) were bought from eBioscience, San Diego, CA, USA; FITC anti-mouse/rat CD29 (#102205) was purchased from Biolegend, San Diego, CA, USA; Caspase 3 Activity Assay Kit, mitochondrial membrane potential assay kit with JC-1, and LDH Cytotoxicity Assay Kit were bought from Beyotime Biotechnology, Haimen, China; Annexin V-FITC/PI Kit was bought from Sangon Biotech, Shanghai, China; artemisinin, dimethyl sulfoxide (DMSO), Alizarin Red S, Oil Red O, β-Glycerophosphate disodium salt hydrate, insulin, and 3-isobutyl-1-methylxantine were received from Sigma (St. Louis, MO, USA); ascorbic acid, dexamethasone, propidium iodide (PI), and indomethacin were purchased from Meilun Biotech Co. Ltd. (Dalian, China); BCA Protein Assay Kit, Cu/Zn-SOD and Mn-SOD Assay Kit with WST-8, Catalase Assay Kit, and Total Glutathione Peroxidase Assay Kit were obtained from Beyotime Institute of Biotechnology (Beyotime, Shanghai, China); anti-Erk1/2 (#9102), anti-phospho-Erk1/2 (#9101), anti-phospho-p90^rsk^ (#9341), anti-phospho-c-Raf (#9421), anti-phospho-CREB (#9198), anti-Bcl-2 (#4223), anti-Bax (#2772), anti-Akt (#9272), anti-phospho-Akt (#9271), and anti-β-actin (#12620) were purchased from Cell Signaling Technology (CST), Woburn, MA, USA; KRAS antibody (#32216) was obtained from SAB, China; anti-GAPDH (sc-32233) was bought from Santa Cruz Biotechnology, Santa Cruz, CA, USA; Clarity Western ECL substrate was purchased from Bio-Rad, Hercules, CA, USA; PD98059 and LY294002 inhibitors were obtained from Merck Millipore, Darmstadt, Germany; siMAPK1, siMAPK3, and siKRAS were purchased from Genepharma, Shanghai, China; fetal bovine serum (FBS) was obtained from GIBCO, Grand Island, NY, USA.

### Animals

The 4-week-old male Sprague Dawley rats were maintained in the Animal Facility of Faculty of Health Sciences, University of Macau (Macau, China). The protocol was approved by the Animal Ethics Committee, University of Macau.

### BMSC isolation and characterization

BMSC isolation and characterization was performed as previously described with minor modification [39]. Briefly, 4-week-old male Sprague Dawley rats were sacrificed by cervical dislocation, and the body was soaked in 70% ethanol alcohol for 10 min. Afterwards, the bone marrow was separated from tibias and femurs and subsequently flushed with MEM alpha containing 10% FBS. The bone marrow was cultured in MEM alpha medium within 10% FBS and 1% penicillin-streptomycin using 75-cm^2^ tissue culture flasks. After cell growth for 24 h, the liquid suspension was replaced with new culture medium. The adherent cells were passaged at 80% confluency, and the cells from only passage three to five were used in the experiments.

Fluorescence-activated cell sorting (FACS) was applied to quantify the level of expression of mesenchymal and hematopoietic markers [3, 40]. Cells were harvested at 80% confluency, centrifuged at 900 rpm for 10 min, supernatant discarded, and cell pellets were resuspended in 3 mL FACS buffer (99% PBS, 1% FBS). Thereafter, the cells at a concentration of 1 × 10^6^/mL were incubated with anti-rat CD 45 FITC, anti-Mouse/Rat CD 90.1 (Thy-1.1) FITC or anti-mouse/rat CD 29 FITC at 4 °C for 30 min, respectively. The expression level of the antigen markers was analyzed through a BD FACSCalibur (BD Biosciences, San Jose, CA). Collected data were analyzed by the software FlowJo 7.6 (Tree Star, Ashland OR, USA).

BMSCs were also identified by investigating the differentiation potential towards osteocytes and adipocytes [41]. For osteogenic differentiation, cells were seeded in 12-well plates at a density of 4 × 10^4^/mL. The culture medium was replaced with osteogenic differentiation medium consisting of DMEM, 10% FBS, 1% penicillin-streptomycin, 1% l-glutamin, 0.1 μM dexamethasone, 50 μM ascorbic acid, and 10 mM glycerol 2-phosphate (BGP) once the cells were approximately 60% confluent. The medium was changed every 3 days. After 21-day culture, cells were gently washed in PBS twice followed by fixation with 4% paraformaldehyde for 20 min at room temperature. Then, cells were stained with 2% Alizarin Red S staining solution for 25 min at room temperature to identify the Ca^2+^ deposits. For adipogenic differentiation, cells were seeded in 12-well plates at a density of 3 × 10^4^/mL. The culture medium was replaced with adipogenic differentiation medium consisting of DMEM, supplemented with 10% FBS, 1% penicillin-streptomycin, 1% l-glutamin, 1 μM dexamethasone, 0.5 mM 3-isobutyl-1-methylxantine (IBMX), 10 μg/mL insulin, and 100 μM indomethacin once the cells were approximately 80% confluent. Fresh medium was changed every 3 days. After 21-day culture, cells were washed with PBS twice and fixed with 4% paraformaldehyde for 20 min at room temperature. Four percent paraformaldehyde was washed with PBS twice followed by being stained with Oil Red O solution for 15 min at room temperature to identify the lipid droplets.

### Assessment of cytotoxicity using MTT assay

The cytotoxicity was analyzed using MTT assay as described [42], with minor modification. Briefly, BMSCs were plated into 96 wells at a density of 5 × 10^4^/mL. For detecting the cytotoxicity of artemisinin or H_2_O_2_, BMSCs were incubated with artemisinin (0.1–100 μM) or H_2_O_2_ (25–800 μM) in serum-free medium for 24 h, followed by the addition of 10 μL MTT (0.5 mg/mL) into each well. After 2-h incubation, medium was discarded and 100 μL DMSO was added in each well. The plate was shaken for 10 min, and then, the optical density (OD) values were read at a wavelength of 570 nm using Infinite M200 PRO Multimode Microplate Reader (Tecan, Männedorf, Switzerland). The viability of living cells was calculated as percentage of control.

### Analysis of cell viability using FACS

FACS was applied to quantify the cell viability of BMSCs by PI staining as described [43], with minor modification. Briefly, BMSCs were plated into 12-well plates at a density of 1 × 10^5^/mL. After appropriate treatment, cells were harvested and washed twice with PBS. Cells were resuspended with the binding buffer into FACS tubes at a density of 2 × 10^5^/mL. PI at the final concentration of 2 μg/mL was added into the resuspended cells followed by incubation 15 min on ice in the dark. The PI fluorescence was determined with BD FACSCalibur using the FL-2 channel. Collected data were analyzed by the software FlowJo 7.6.

### Measurement of cell viability using LDH release assay

LDH is a cytosolic enzyme present in the cell cytoplasm compartment and released into the extracellular space when the plasma membrane is damaged. Therefore, the LDH level is considered as an indicator of cell damage and can be quantified by the enzymatic reaction [44] measuring necrotic cell death [45]. Briefly, BMSCs were plated into 96 wells (5 × 10^4^ cells/mL) and incubated for 24 h. After appropriate treatments, the supernatant was collected for LDH measurement at 560/590 nm using Infinite M200 PRO Multimode Microplate Reader according to the instructions of LDH Cytotoxicity Assay Kit. The percentage of LDH release (%) was calculated compared to control untreated group.

### Measurement of cell viability using Hoechst 33342 staining

Hoechst 33342 staining assay labels nuclear DNA and allows visualization of the nucleus in the interphase and chromosomes in the mitotic living cells. To analyze living BMSCs cells, Hoechst 33342 staining assay was employed [46]. In brief, BMSCs were plated into 24 wells (2.5 × 10^4^ cells/well). After treatments, cells were fixed in 4% formaldehyde for 20 min (25 °C). Subsequently, 5 μg/mL Hoechst 33342 dye solution (50 μl/well) was used to stain cells for 10 min. After two washings, the nuclei of BMSCs were visualized by EVOS FL Imaging System (Thermo Fisher Scientific, Waltham, MA, USA). Apoptotic dying cells were identified as the cells with blue fragmented, condensed nuclei, and the percentage of apoptotic BMSCs was calculated as from total number of cell population. The percentage of apoptotic cells was analyzed by Image J software (https://imagej.nih.gov/ij/download.html, National Institute of Health, Bethesda, MD, USA).

### Measurement of the mitochondrial membrane potential (ΔΨm)

JC-1 assay was performed to measure ΔΨm as previously published [36] and using the JC-1 mitochondrial membrane potential assay kit. In short, BMSCs were plated (5 × 10^4^ cells/mL) in 6 wells and after appropriate drug treatments; the detection of ΔΨm was carried out according the guidelines of JC-1 kit. Images were taken by using EVOS FL Imaging System. The ratio (%) of fluorescence red/green fluorescence intensity was calculated by Image J software, and the value was calculated relative to the control group.

### Measurement of apoptosis using Annexin V-FITC/PI assay

The apoptotic rates were analyzed as described [47] with slight modification, by using the FACS methodology using a protocol recommended in the Annexin V-FITC/PI Kit. Briefly, BMSCs were plated into 6 wells (2 × 10^5^ cells/mL). After the appropriate treatments, the cells were harvested and washed with the binding buffer. Then, cells were suspended in 195 μL binding buffer at a cell density of 2 × 10^5^ cells/mL, incubated with Annexin V-FITC (10 μg/mL, 5 μL) and incubated at 25 °C for 15 min in dark. At the end of incubation, BMSCs were washed with the binding buffer (200 μL) and centrifuged at 1000 rpm for 5 min. Cells were suspended in 190 μL binding buffer with 10 μL PI (20 μg/mL). The samples were acquired in BD FACSCalibur, and the collected data were analyzed by the software FlowJo 7.6. The apoptosis rate was expressed as the percentage of Annexin V-positive cells.

### Measurement of apoptosis using Caspase 3 activity assay

Caspase 3 activity was measured using the Caspase 3 Activity Assay Kit [48, 49] according to manufacturer’s protocol. In brief, BMSCs were plated into 6 wells (5 × 10^4^ cells/mL) and treated as indicated. Culture medium and adherent cells were collected and centrifuged for 5 min (4 °C, 600*g*). Collected cells were resuspended and incubated in the lysis buffer (supplied with the kit) on ice for 15 min before being centrifuged for 12 min (4 °C, 18000*g*). The cell lysate supernatant was collected. The final 100 μL reaction mixture contained 40 μL assay buffer (supplied with the kit), 50 μL cell lysate supernatant, and the 10 μL Caspase 3 substrate Ac-DEVD-pNA (2 mM) (supplied with the kit). The mixture was incubated at 37 °C for 120 min, and then, Caspase 3 activity was measured at 405 nm using the Infinite M200 PRO Multimode Microplate Reader. The percentage of Caspase 3 activity was calculated compared to control group.

### Measurement of oxidative stress

ROS in BMSCs were evaluated by using CellROX® Deep Red Reagent as described [34]. After appropriate treatments, medium was replaced by medium with 5 μM CellROX® Deep Red reagent and the cells were incubated at 37 °C without light for 1 h. Then, BMSCs were washed for three times with PBS and the images of the cells were taken by the EVOS FL Imaging System at the excitation and emission wavelengths at 640 nm and 665 nm separately. Semi-quantification of the oxidative level was calculated by using the software Image J and the percentage of oxidative level (%) was calculated compared to the control group.

### Measurement of SOD, CAT, and GPx activities

Superoxide dismutase (SOD), catalase (CAT), and glutathione peroxidase (GPx) enzymatic activities were determined by using Cu/Zn-SOD and Mn-SOD Assay Kit with WST-8, Catalase Assay Kit, and Total Glutathione Peroxidase Assay Kit (Beyotime, Beijing, China) following the manufacturer’s instructions. Briefly, SOD activity determination was based on the inhibition of the superoxide radical-dependent cytochrome C reducing measured at a wavelength of 450 nm using Infinite M200 PRO Multimode Microplate Reader. CAT activity determination was based on the reducing absorbance at 520 nm due to the ability of scavenging H_2_O_2_, and the enzyme activity was converted by the speed of H_2_O_2_ consumption based on a standard curve obtained by the scalar units testing. GPx activity was determined according to that the speed of NADPH decrease was proportional to GPx activity measured at 340 nm using Infinite M200 PRO Multimode Microplate Reader. Enzyme activities were expressed as a percentage of control.

### Western blotting

Western blotting was performed as described [50]. In brief, cells were rinsed by PBS and lysed in the RIPA buffer at 4 °C. Concentration of proteins was determined using BCA protein kit. The proteins were separated by polyacrylamide gel electrophoresis and electro-transferred to nitrocellulose (NC) membranes (Millipore, Billerica, MA, USA). Membranes were incubated with 3% (w/v) bovine serum albumin (BSA) in TBST (TBS with 0.1% Tween 20) for 1 h at the room temperature and incubated with the corresponding primary antibodies at 4 °C for 10 h. Thereafter, the membranes were washed with TBST several times and probed at room temperature for 1 h with secondary antibodies conjugated with horseradish peroxidase. The membranes were washed several times with TBST to remove the unbound secondary antibodies and then visualized using Clarity Western ECL substrate, as described in the instructions of the manufacturer.

The phosphorylation of Erk1/2, c-Raf, p90^rsk^, CREB, and Akt was detected by western blotting using the respective anti-phospho-antibodies. Expression of KRAS, Bcl-2, and Bax were also measured. Blots were stripped and then reprobed with anti-total Erk1/2 antibodies to assess that equal amounts of p-Erk1/2. Blots were also stripped and reprobed with anti-GAPDH or β-actin antibodies, respectively, for different approaches of normalization. The intensity of the protein bands was analyzed by the Image J software.

### Statistical analysis

All experiments were performed in triplicate, and data was expressed as the mean ± standard deviation (SD). One-way ANOVA followed by Tukey’s multiple comparison was used in the statistical analysis with the aid of the software Graph Pad Prism 5.0 (Graph Pad Software Inc., USA), and the value *p* < 0.05 was considered as statistically significant.

## Results

### Characterization of primary cultures of rat BMSCs

**Fig. 1:**
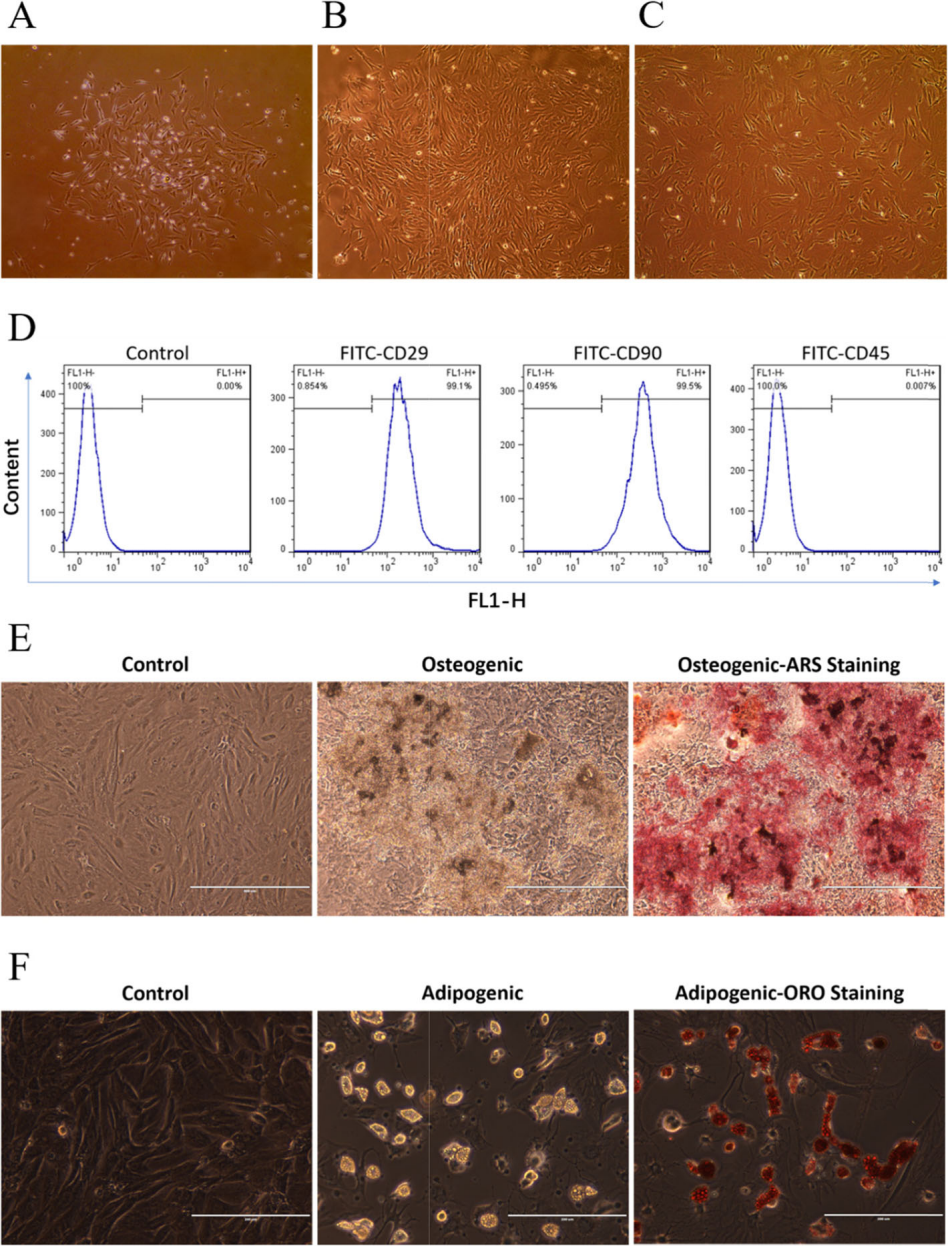
Rat BMSC culture morphology (**a**–**c**), CD marker expression being evaluated by FACS (**d**), osteogenic and adipogenic differentiation induction (**e**, **f**). **a** The fourth day after isolation. **b** Passage 3. **c** Passage 5. **d** The expression levels of mesenchymal markers CD29, CD90, and hematopoietic marker CD45. **e** Differentiation potential to osteocytes. Cells were cultured in osteogenic differentiation medium for 21 days followed by the Alizarin Red S (ARS) staining. The representative images were presented here. Control, cultured with normal medium; osteogenic, cultured with osteogenic differentiation medium. **f** Differentiation potential to adipocytes. Cells were cultured in adipogenic differentiation medium for 21 days followed by the Oil Red O (ORO) staining. The representative images were presented here. Control, cultured with normal medium; adipogenic, cultured with adipogenic differentiation medium

Primary cultured rat BMSCs were isolated from BM [51] by their adhesion to the tissue culture surfaces, consisted of a heterogeneous cell population with a dominant spindle-shaped morphology and able to generate fibroblast-like colonies (Fig. 1a). Cells were passaged when reached about 80% confluency at 9 to 11 days in culture. Adherent cells of passage three (Fig. 1b) and passage five (Fig. 1c) showed typical spindle-shaped cell morphology [52] and were therefore used in all experiments. For further evaluation of the BMSCs phenotype, the cell-surface antigens were then analyzed by FACS which confirmed the expression of typical BMSC mesenchymal characteristic markers, i.e., CD29 (99.1% of the cell population) and CD90 (99.5% of cell population), as well as the absence of the early hematopoietic CD45 marker (less than 0.007% of cell population expressed this marker) [53, 54].

Characterization of osteogenic differentiation was performed by the determination of Ca^2+^ deposits using Alizarin Red S staining. The undifferentiated BMSCs (Fig. 1e, left) were cultured in normal medium. Accumulation of Ca^2+^ deposits started to be observed at the 10th day and became more at the 21th day in osteogenic differentiation medium (Fig. 1e, middle). In Alizarin Red S staining, Ca^2+^ deposits were stained red (Fig. 1e, right). Characterization of adipogenic differentiation was performed by Oil Red O staining. The undifferentiated BMSCs were cultured in normal medium and did not show any adipocyte characteristics (Fig. 1f, left). After 21-day cultivation in adipogenic differentiation medium, the lipid droplets were observed (Fig. 1f, middle), and after Oil Red O staining, lipid droplets were stained red (Fig. 1f, right).

### Artemisinin protection towards H_2_O_2_-induced necrotic cell death

**Fig. 2:**
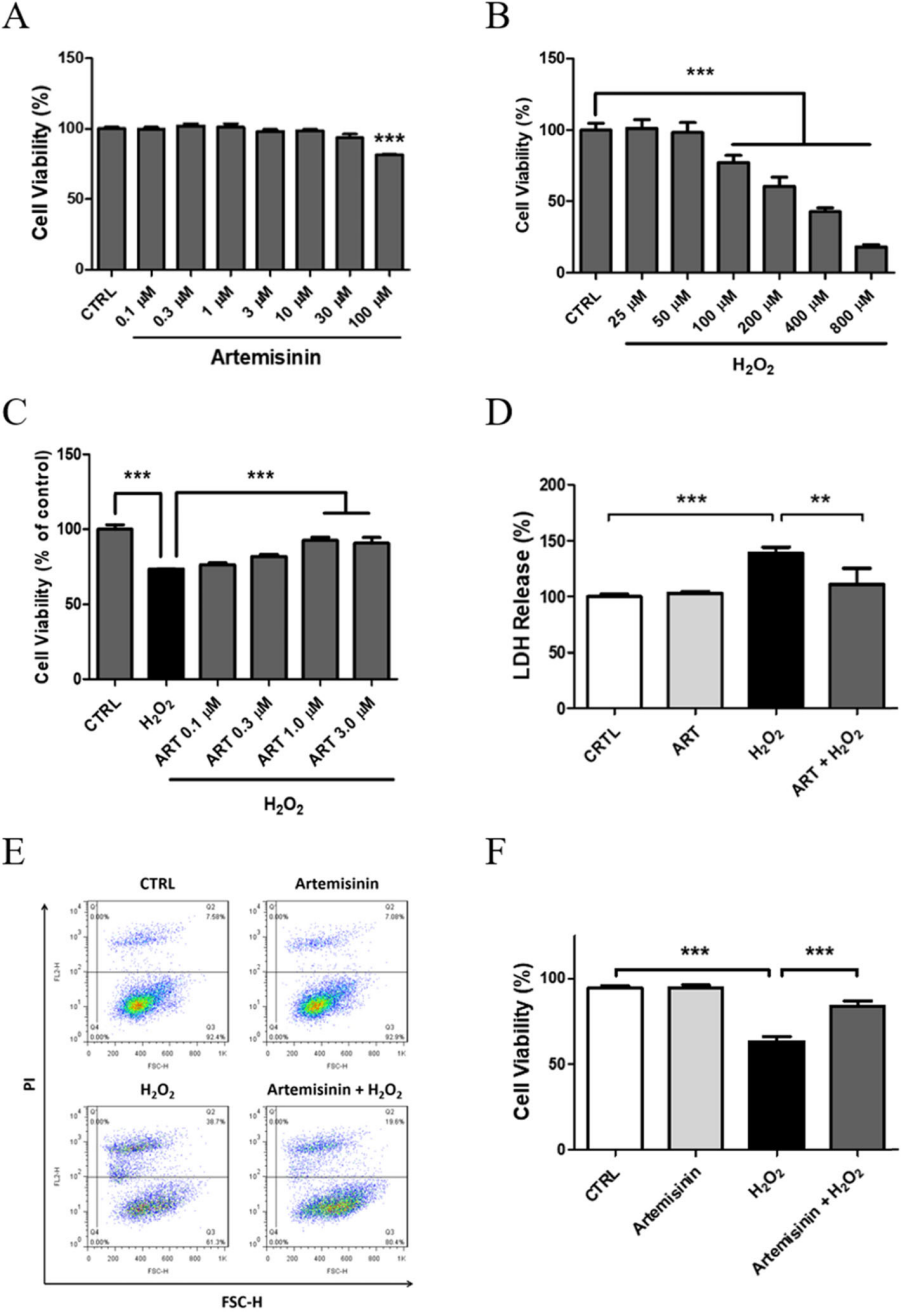
Artemisinin cytoprotective effects towards H_2_O_2_-induced necrosis in BMSCs. **a** Pretreatment for 24 h with different concentrations of artemisinin or DMSO (0.1%, vehicle control); cell viability was measured by MTT assay (*n* = 3). **b** BMSCs were treated with different concentrations of H_2_O_2_ for 24 h, and the cell viability was measured by MTT assay (*n* = 3). **c** BMSCs were pretreated with different concentrations of artemisinin (ART) or DMSO (1%, vehicle control-CTRL) for 1 h before exposure to 200 μM H_2_O_2_ for another 24 h; the cytotoxicity was detected by MTT assay (*n* = 3). **d** BMSCs were pretreated with 1.0 μM artemisinin or DMSO (0.1%, vehicle control) for 1 h and then exposed to H_2_O_2_ for another 24 h; necrotic cell death was measured by LDH release (*n* = 3). **e** Cells were pretreated with 1.0 μM artemisinin or DMSO (0.1%, vehicle control) for 1 h and then exposed to 200 μM H_2_O_2_ for another 24 h; the cell viability was analyzed by PI staining, detected by FACS, and presented as % of the total number of cells (**f**) (*n* = 3). **a**, **b**, **f** ***p < 0.005 compared to control group; **c**, **d** **p < 0.01, ***p < 0.005. CTRL, control; ART, artemisinin; H_2_O_2_, exposed to hydrogen peroxide only; ART + H_2_O_2,_ treated with artemisinin followed by exposure to hydrogen peroxide

To investigate the protective effect of artemisinin on H_2_O_2_-induced cytotoxicity in BMSCs, we first analyzed the range of artemisinin concentrations which are not cytotoxic (Fig. 2a). BMSC cultures were treated with different concentrations of artemisinin (0.1–100 μM), for 24 h, and the cell viability was measured using the MTT assay. Treatment with 0.1 to 30 μM was safe, while cell viability decreased by 20% upon treatment with 100 μM artemisinin (Fig. 2a). Therefore, in all future experiments, the maximal concentration of artemisinin used was 30 μM. In the next step, to evaluate the cytotoxicity of H_2_O_2_, BMSCs were treated with different concentrations of H_2_O_2_ for 24 h. Figure 2b indicates that cell viability was decreased in a concentration-dependent manner. Two hundred micromolar H_2_O_2_ decreased by 39.5% cell viability, and therefore, this concentration was chosen in all future experiments evaluating the potential cytoprotective effect of artemisinin.

In the next step, to investigate the protective effect of artemisinin, BMSCs pretreated with artemisinin or DMSO (0.1%, the vehicle control) at concentrations from 0.1 to 3.0 μM for 1 h were exposed to 200 μM H_2_O_2_ for 24 h and the viability was analyzed by MTT assay (Fig. 2c). Cell viability of H_2_O_2_ group decreased significantly compared to the control group, and artemisinin at concentrations of 1.0 and 3.0 μM abrogated the H_2_O_2_-induced death. The cytoprotective effect of artemisinin against H_2_O_2_-induced death was also showed in FACS analysis using PI staining (Fig. 2e, f). In the fourth step, using the LDH assay to measure necrotic cell death (Fig. 2d), we also found that artemisinin abrogated H_2_O_2_-induced LDH release, confirming the cytoprotective effect measured in Fig. 2c and e, f.

### Artemisinin protection towards H_2_O_2_-induced apoptotic cell death

**Fig. 3:**
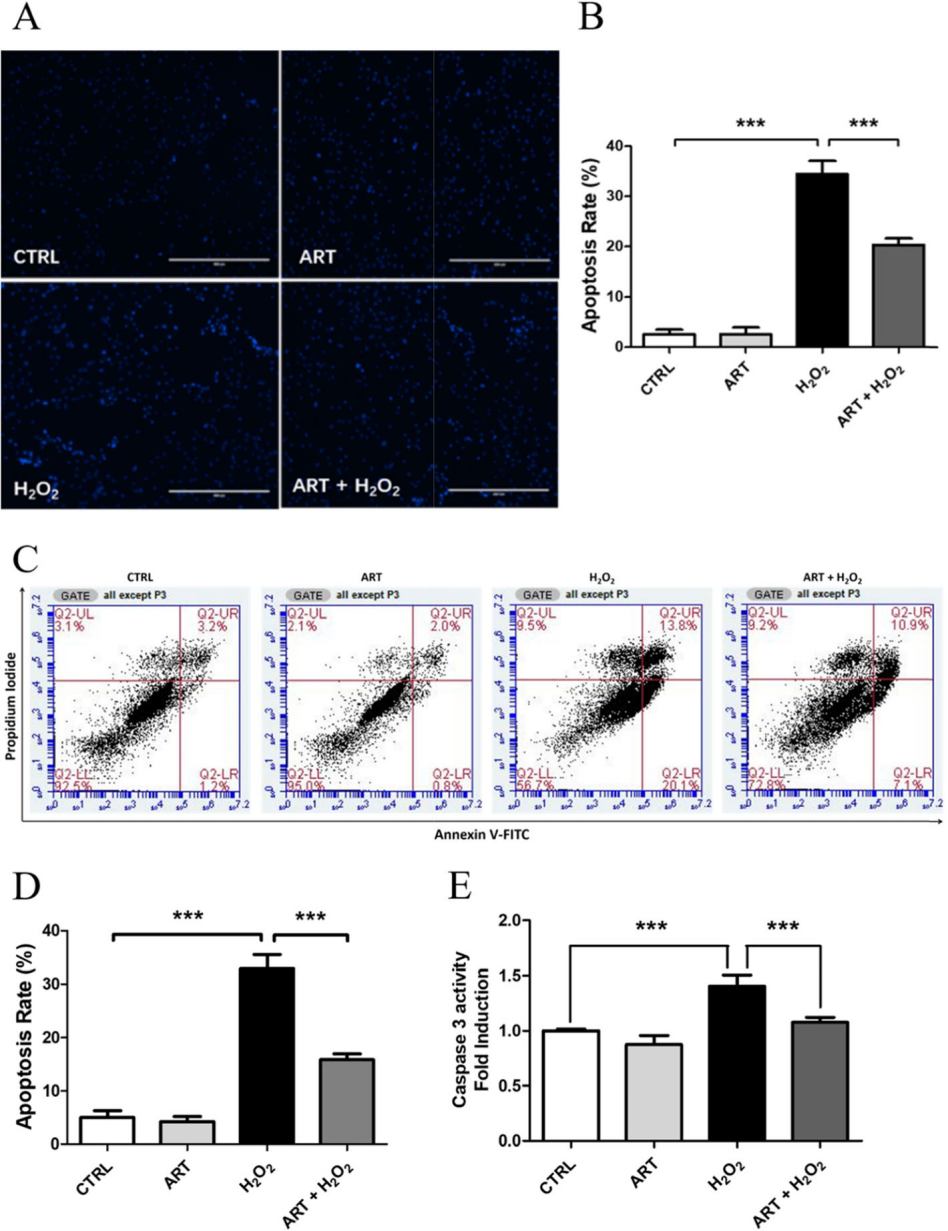
Artemisinin cytoprotective effects towards H_2_O_2_-induced apoptosis in BMSCs. After the pretreatment with artemisinin (1.0 μM) or DMSO (0.1%, vehicle control) for 1 h, BMSCs were exposed to 200 μM H_2_O_2_ for another 24 h. **a** Cell apoptosis was measured by using nuclear DNA Hoechst staining observed by fluorescence microscopy to detect changes in cell nuclei y (*n* = 3). **b** The apoptotic and total cells were analyzed by Image J software, and the apoptosis rate was presented as % of the total number of nuclei (*n* = 3). **c** The apoptotic cell death of BMSCs was analyzed by Annexin V-FITC/PI staining, detected by FACS (*n* = 3), and quantified by the apoptosis rate (**d**) (*n* = 3). **e** The activity of caspase 3 was measured by caspase 3 activity assay (*n* = 3). ***p < 0.005. CTRL, control; ART, artemisinin; H_2_O_2_, exposed to hydrogen peroxide only; ART + H_2_O_2_, treated with artemisinin followed by exposure to hydrogen peroxide

To further verify the cytoprotective effects of artemisinin, apoptotic cell death assays using nuclear DNA Hoechst staining observed by fluorescence microscopy to detect changes in cell nuclei and Annexin V-FITC/PI staining detected by FACS were performed. After BMSC pre-treatment with 1.0 μM artemisinin for 1 h, the number of cells with bright blue fluorescence were reduced remarkably, indicating that artemisinin can markedly decrease the number of apoptotic cells and nuclear condensation caused by H_2_O_2_ (Fig. 3a, b). The results of Annexin V-FITC/PI staining detected by flow cytometry revealed (Fig. 3c, d) that the H_2_O_2_ treatment group had a significantly higher rate of apoptosis (32.90 ± 4.60%) compared with the control group (5.03 ± 2.17%) (*P* < 0.05). Treatment with artemisinin significantly protected the cells towards H_2_O_2_-induced apoptosis and significantly reduced the rate of apoptotic cells (*P* < 0.01; 15.81 ± 1.94%). In line with these findings, direct measurements of caspase 3 activity, presented in Fig. 3e, indicate that artemisinin (1.0 μM) treatment for 1 h abrogated H_2_O_2_ activation of caspase 3.

### Artemisinin protection on mitochondria

**Fig. 4:**
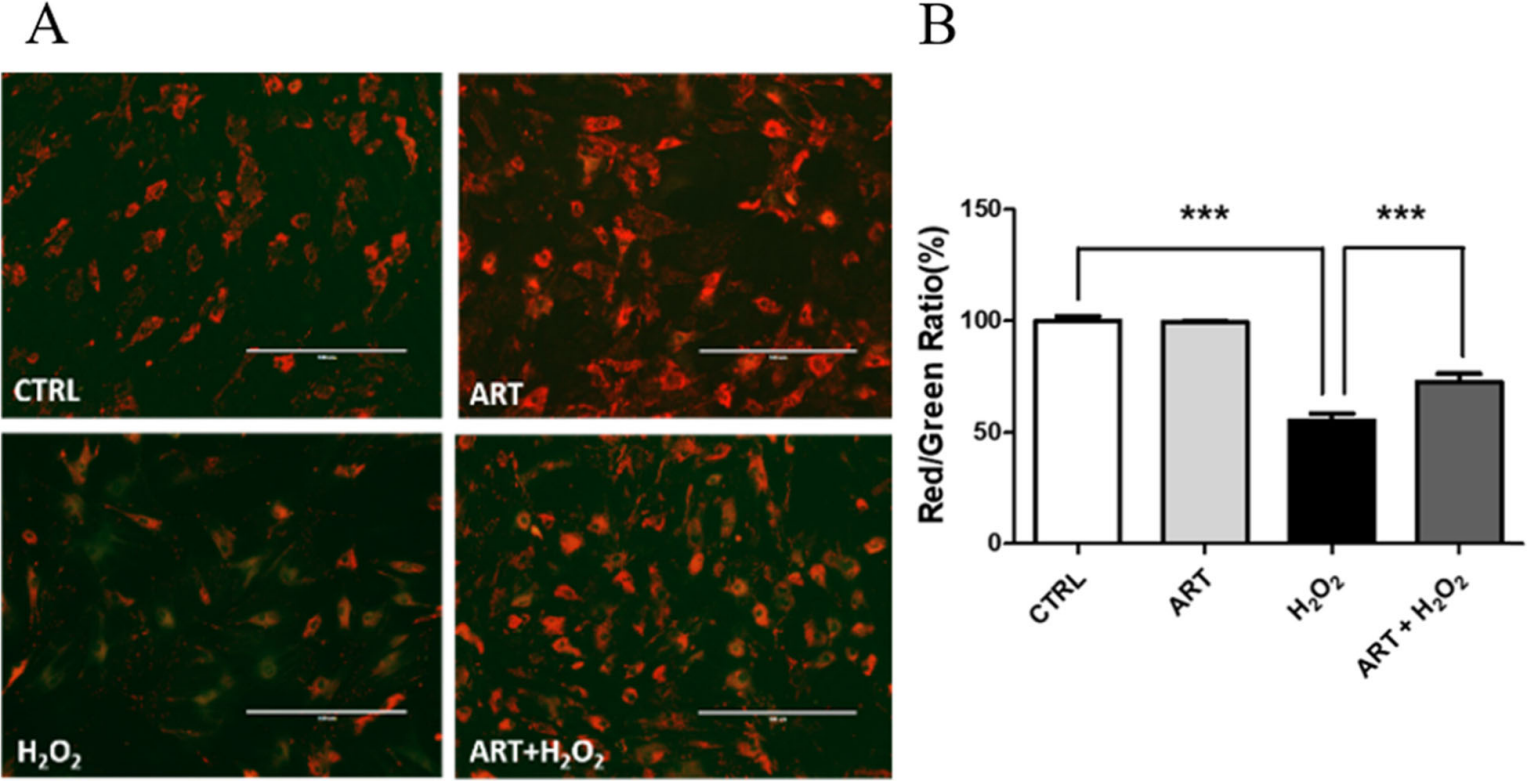
Artemisinin attenuated H_2_O_2_-induced ΔΨm reduction. BMSCs were pretreated with 1.0 μM artemisinin or DMSO (0.1%, vehicle control) for 1 h, then exposed to 200 μM H_2_O_2_ for another 24 h. **a** ΔΨm was analyzed using JC-1 assay (*n* = 3; scale, 100 μm). The decline of the membrane potential was reflected by the shift of fluorescence from red to green indicated by JC-1. **b** The quantitative data of the red/green ratio (*n* = 3). ***p < 0.005. CTRL, control; ART, artemisinin; H_2_O_2_, exposed to hydrogen peroxide only; ART + H_2_O_2_, treated with artemisinin followed by exposure to hydrogen peroxide

Mitochondria in eukaryotic cells are the major components of respiration and play a critical role in the defense towards oxidative stress-induced insults. Maintaining the Ψm is important to ensure the scavenging efficiency of ROS, and to confer cytoprotection towards apo-necrotic events induced by excessive ROS [55]. JC-1 is a Ψm-sensitive dye, aggregates in mitochondrial matrix, and exhibits a red fluorescence in the healthy cells. When Ψm is reduced, JC-1 is converted to the monomer state exhibiting a green fluorescence. Therefore, the effects of artemisinin on mitochondrial membrane potential of BMSCs were investigated using the fluorescent dye JC-1. As expected, 200 μM H_2_O_2_ for 24-h treatment resulted in a noticeable reduction in Ψm of BMSCs, whereas artemisinin markedly increased the Ψm, as identified by fluorescence microscopy (Fig. 4a). In addition, the ratio of red/green fluorescence intensity was significantly decreased by H_2_O_2_ compared with the control group, but the effect was reversed by artemisinin (Fig. 4b). These findings suggest that artemisinin may exert beneficial effects on mitochondrial function.

### Artemisinin decreased the production of ROS but increased the activities of SOD, CAT, and GPx in H_2_O_2_-treated BMSCs

**Fig. 5:**
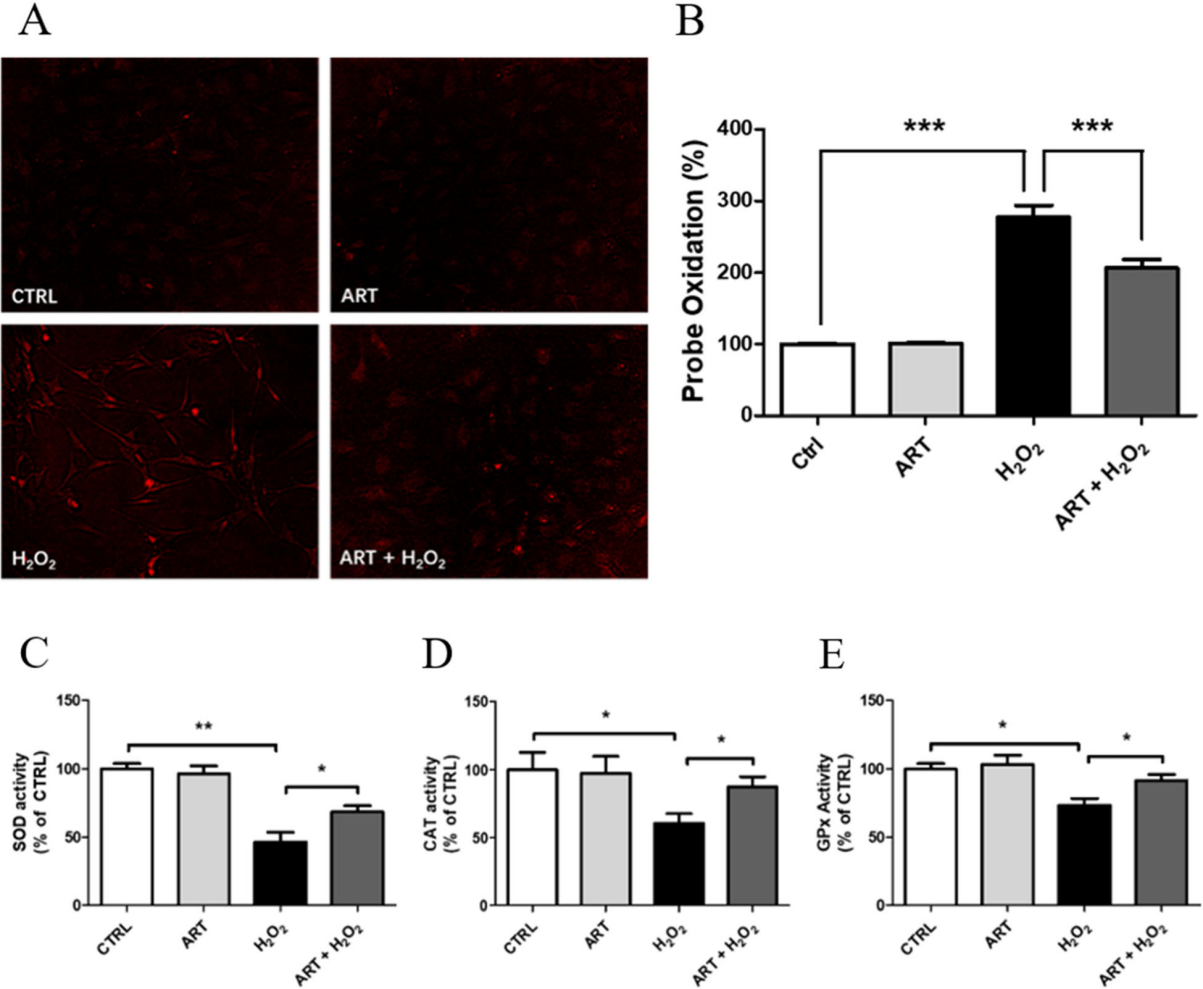
Artemisinin decreased intracellular ROS level but increased SOD, CAT, and GPx activities treated with H_2_O_2_. BMSCs were pretreated with 1.0 μM artemisinin or 0.1% DMSO (vehicle control) for 1 h, followed by 24-h exposure to 200 μM H_2_O_2_. **a** Fluorescent visualization of ROS production in rat BMSCs using the CellROX® Deep Red fluorescence imaging. Cells were incubated with reagent for 60 min after treatments. H_2_O_2_-treated cells displayed red fluorescence, indicating increased ROS production (*n* = 3). **b** The quantitative analysis of the intracellular ROS level. Bars show the average percent of fluorescent positive cells out of total cell number (*n* = 3). ***p < 0.005. **c** The relative SOD activity (*n* = 3), *p < 0.05, **p < 0.01. **d** The relative CAT activity (*n* = 3), *p < 0.05. **e** The relative GPx activity (*n* = 3), *p < 0.05. CTRL, control; ART, artemisinin; H_2_O_2_, exposed to hydrogen peroxide only; ART + H_2_O_2_, treated with artemisinin followed by exposure to hydrogen peroxide

ROS are important mediators of H_2_O_2_-induced cell death [56, 57]. In order to evaluate the effect of artemisinin, on ROS levels upon H_2_O_2_-induced BMSC death, the cultures were pretreated with 1 μM artemisinin for 1 h followed by exposure to 200 μM H_2_O_2_ for 24 h (Fig. 5). The data clearly indicates that intracellular ROS level significantly increased upon exposure to H_2_O_2_ (276.69 ± 17.19%) while pretreatment with artemisinin significantly attenuated the ROS production (206.61 ± 11.86%) induced by H_2_O_2_ (Fig. 5a, b). To investigate whether the antioxidant enzyme activities are mediated by artemisinin, we measured the activities of SOD, CAT, and GPx in H_2_O_2_-treated BMSCs. When BMSCs were treated with 200 uM H_2_O_2_, the activities of SOD, CAT, and GPx were significantly decreased compared with control, whereas treatment with artemisinin significantly increased the activities of these three enzymes compared with H_2_O_2_ (Fig. 5c–e).

### Artemisinin stimulated activation/phosphorylation of Erk1/2 is involved in the protective effect

**Fig. 6:**
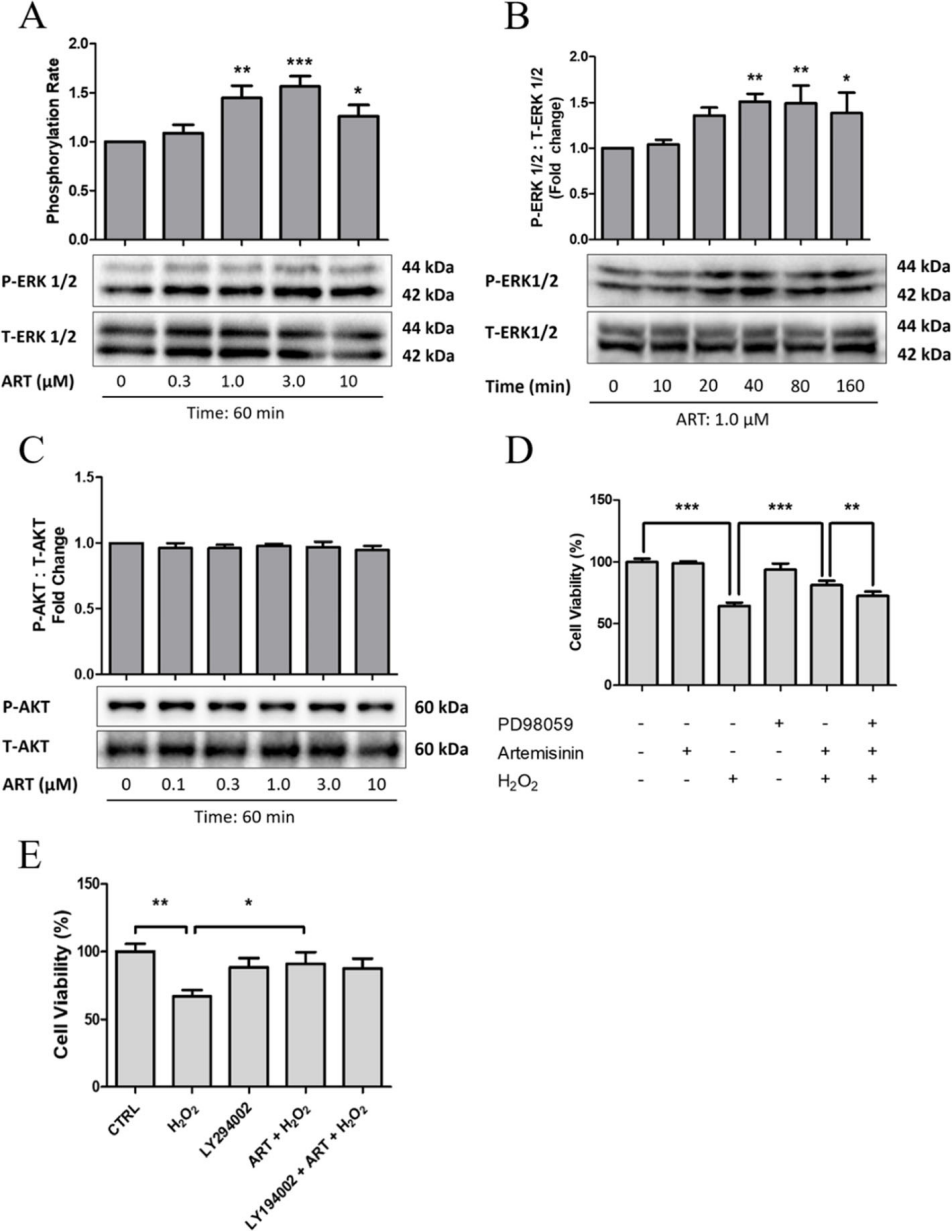
Artemisinin activation of Erk1/2 but not PI3K is involved in the protective effect. **a**–**c** BMSCs were pretreated with various concentrations of artemisinin for 1 h or 1.0 μM artemisinin for up to 160 min. Cell lysates were submitted for western blotting to measure phosphorylation (P) and total level (T) of Erk1/2 and Akt (*n* = 3). **d**, **e** BMSCs were preconditioned with 10 μM PD98059 or LY294002 for 30 min and then treated with 1.0 μM artemisinin for 1 h followed by exposure to 200 μM H_2_O_2_ for 24 h, and thereafter, the cell viability was determined using MTT assay (*n* = 3). **a**, **b** *p < 0.05, **p < 0.01, ***p < 0.005 versus control (0 μM in **a**, 0 min in **b**); **d**, **e** *p < 0.05, **p < 0.01, ***p < 0.005 versus control

**Fig. 7:**
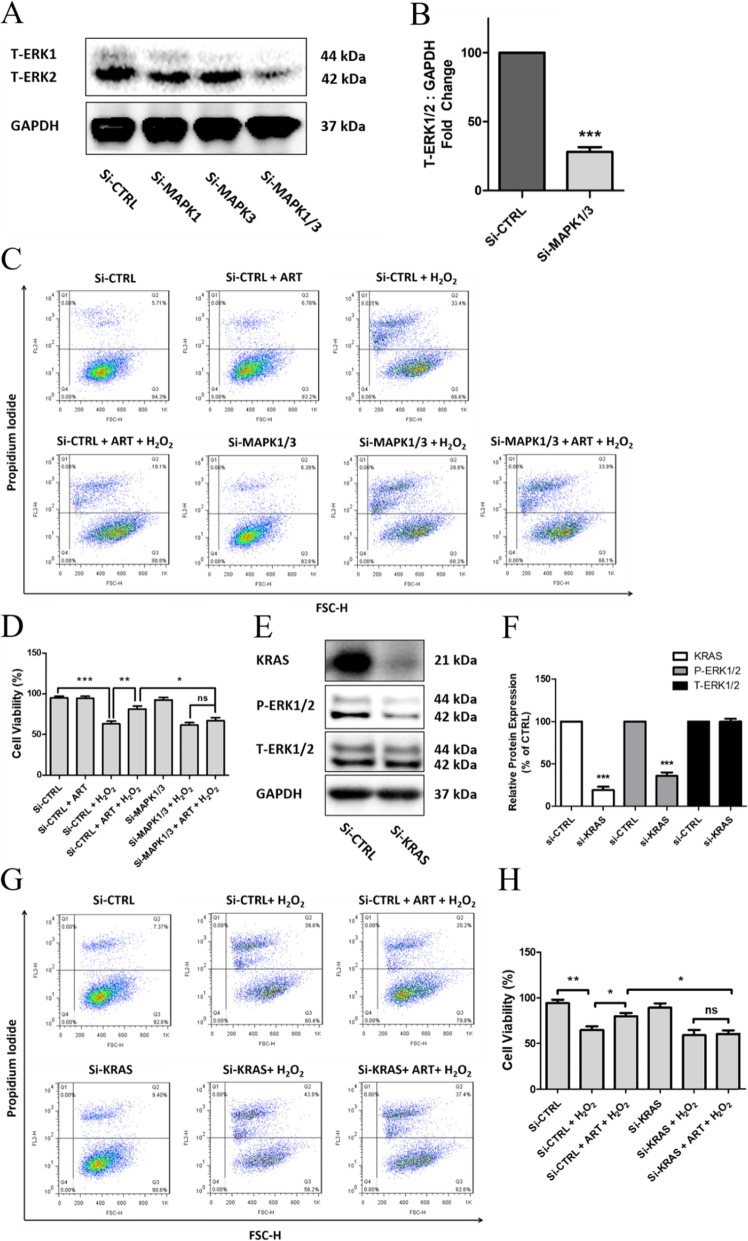
Decreased expression of Erk1/2 and KRAS by siRNA attenuated the protective effect of artemisinin. **a** BMSCs were transfected with 80 nM siMAPK1 (Erk2) or with 80 nM siMAPK3 (Erk1), or co-transfected with 80 nM siMAPK1 and siMAPK3 for 48 h; the expression of T-Erk1, T-Erk2, and GAPDH were detected by western blotting (*n* = 3). **b** The quantitative analysis of T-Erk1/2 relative expression using Image J software (*n* = 3). ***p < 0.005 versus control group. **c** BMSCs were co-transfected with 80 nM siMAPK1 and siMAPK3 as above and pretreated with 1.0 μM artemisinin for 1 h followed by exposure to 200 μM H_2_O_2_ for another 24 h, and the cell viability was analyzed by FACS using PI staining and quantified (**d**) (*n* = 3). *p < 0.05, **p < 0.01, ***p < 0.005; ns, not significant. **e** BMSCs were transfected with 40 nM siKRAS for 72 h; the expressions of KRAS, p-Erk1/2, and t-Erk1/2 were detected by Western blotting. **f** The quantitative analysis of KRAS, p-Erk1/2, and t-Erk1/2 was performed using Image J software (*n* = 3). ***p < 0.005. **g** The cell viability was determined by FACS using PI staining and quantified (**h**) (*n* = 3). *p < 0.05, **p < 0.01, ns, not significant

In previous studies we reported that the Erk1/2 signaling pathway mediate the protective effects of artemisinin in rat PC 12 dopaminergic neuronal and human retinal pigment epithelial cells [58]. We therefore sought to investigate whether this pathway was also involved in the protective effects of artemisinin in BMSCs. To verify this hypothesis, BMSCs were treated for different time periods or with various concentrations of artemisinin and culture extracts were evaluated by western blotting to measure the phosphorylation activities of Erk1/2 and Akt. The phosphorylation levels of Erk1/2 were increased by 1.5-fold after 1-h treatment with either 1 or 3 μM artemisinin (Fig. 6a). This phosphorylation was maximal at 40–60 min and thereafter declined (Fig. 6b). By contrast, treatment with artemisinin for 1 h, up to a concentration of 10 μM, did not affect Akt phosphorylation (Fig. 6c). To gain a further insight into the role of the Erk1/2 pathway in the protective effect of artemisinin on BMSCs, the cells were preconditioned with either 10 μM PD98059 (Erk1/2 inhibitor, Fig. 6d) or LY294002 (PI3K inhibitor, Fig. 6e) for 30 min and thereafter treated with 1.0 μM artemisinin for 1 h followed by exposure to 200 μM H_2_O_2_ for 24 h, and at the end of experiment, the cell culture viability was measured using the MTT assay. The results presented in Fig. 6d, e clearly indicate that Erk1/2 but not PI3K inhibition significantly reduced artemisinin’s protective effect towards H_2_O_2_ insult. Similarly, the silence of Erk1/2 by siMAPK1 (Erk2) and siMAPK3 (Erk1) also blocked the protective effect of artemisinin in BMSCs (Fig. 7a–d). Furtherly, the phosphorylation of Erk1/2 was significantly blocked when the upstream gene KRAS was knocked down by siKRAS, and the protection of artemisinin was also attenuated (Fig. 7e–h).

**Fig. 8:**
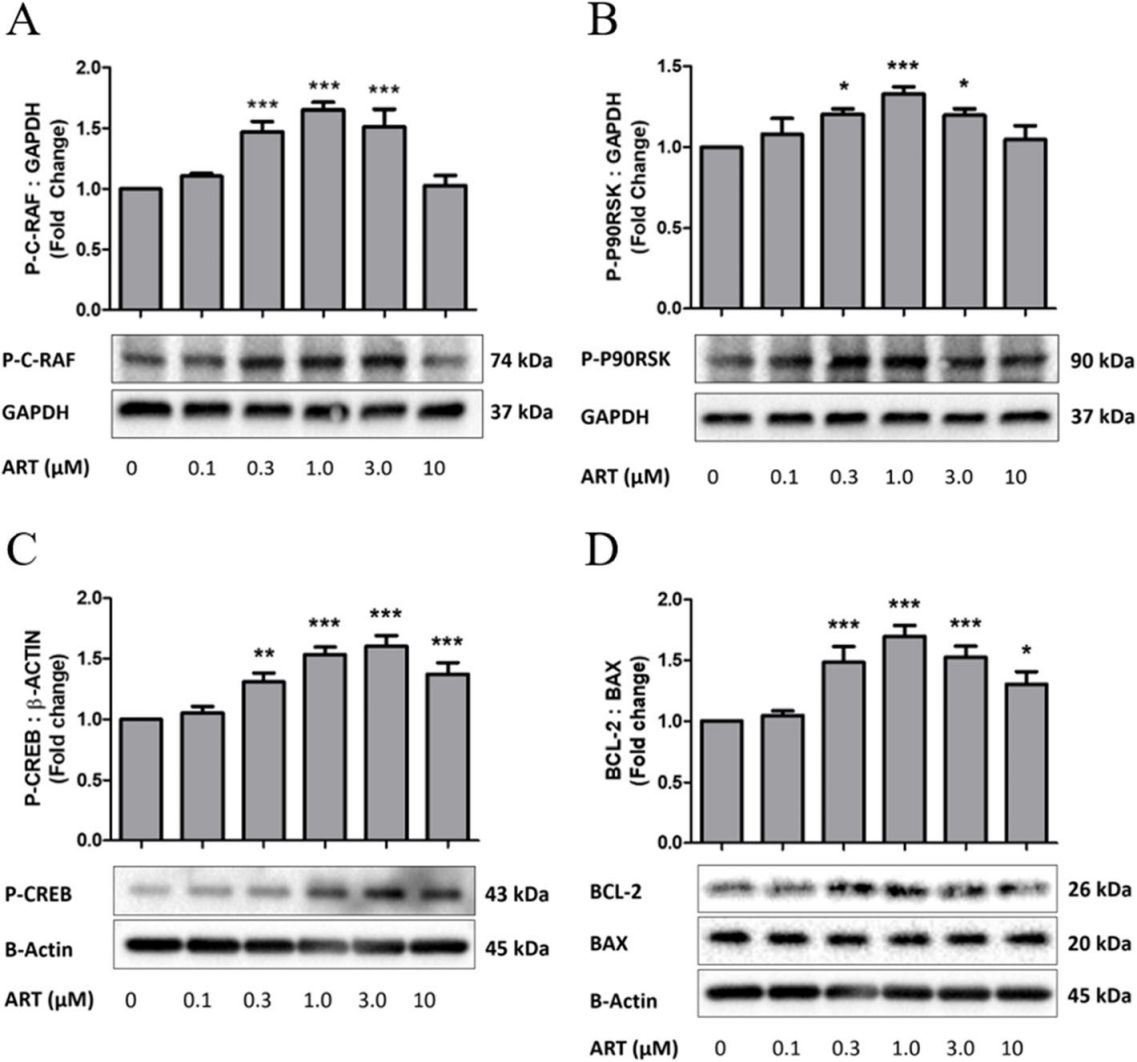
Artemisinin activation of c-RAF (**a**), p90^RSK^ (**b**), CREB (**c**) phosphorylation and increased expression level of Bcl-2 (**d**). BMSCs were treated with various concentrations of artemisinin for 1 h, and then, culture lysates were submitted for western blotting with respective antibodies (*n* = 3); *p < 0.05; **p < 0.01; ***p < 0.005 versus control group (0 μM)

To further strength the involvement of Erk1/2 signaling pathway in artemisinin protective effect on BMSCs, we sought to investigate whether the phosphorylation of c-Raf (upstream of Erk1/2), p90^RSK^ (downstream of Erk1/2), CREB (downstream of Erk1/2), and Bcl-2 (a typical anti-apoptotic protein) is also affected by artemisinin treatment of the cultures (Fig. 8). For this purpose, BMSCs were treated with various concentrations of artemisinin for 1 h, and then, the culture extracts were evaluated by western blotting for phosphorylation of these proteins. At concentrations of 0.3, 1, and 3 μM artemisinin, the phosphorylation of all these signaling proteins was increased by 1.2–1.7-fold compared to control (Fig. 8). Cumulatively, these results propose that c-Raf-Erk1/2-p90^RSK^-CREB signaling pathway was activated by artemisinin in BMSCs.

## Discussion

In the present study, we found that artemisinin protected primary cultured rat BMSCs towards H_2_O_2_-induced cell death. However, to the best of our knowledge, this is the first time that the antimalarial compound, artemisinin, was characterized for protective effects in insulted stem cells. Present findings suggest that activation of c-Raf-Erk1/2-p90^RSK^-CREB signaling pathway was involved in the protective action of artemisinin towards oxidative stress injury. Transplantation of BMSCs becomes a potential therapy for several ischemic diseases, but the oxidative stress injury of BMSCs limits the survival of the transplanted cells and as a result failure in therapy. Present findings therefore may offer a method to protect BMSCs against oxidative stress.

At physiological levels, ROS might serve as a second messenger in the signaling pathways of proliferation and differentiation of cells in general and stem cells in particular [59]. It is also known that low-level of cellular ROS partly promotes proliferation, growth, and survival of the cells [60, 61]. However, at pathological ROS levels, they contribute to apoptotic BMSC death and restrain their differentiation [62]. It was reported that during oxidative stress, telomere length of mesenchymal stem cells were shortened, cells underwent replicative senescence, and their differentiation abilities was decreased [63, 64]. The increase levels of ROS promoted phosphorylation of c-Jun N-terminal kinases which translocated from the cytoplasm to mitochondria resulting with caspase 3 activation followed by apoptosis [11]. Indeed, present findings (Fig. 3) support this line of research and further emphasize that artemisinin conferred protection by significantly suppressing ROS levels, increasing SOD, CAT, and GPx activities and decreasing caspase 3 activation induced in rat BMSCs exposed to H_2_O_2_ insult (Fig. 5). Mitochondria play a crucial role in the growth, survival, apoptosis, and some other fundamental cell functions [65]. Pretreatment of BMSCs with artemisinin significantly suppressed H_2_O_2_-induced collapse of ΔΨm (Fig. 4b). Cumulatively, these results provide circumstantial evidences on an apparent relationship between antioxidant activity, mitochondrial membrane potential stabilization, and cytoprotective effects of artemisinin.

Artemisinin pretreatment of the BMSC cultures provided efficient cytoprotection towards H_2_O_2_-mediated apo-necrotic cell death by blocking or attenuating the increase of caspase 3 activity, LDH release, and nuclei condensation. These effects were again temporally correlated with the activation of Erk1/2. Erk1/2 can be activated by a variety of extracellular stimuli, such as serum, growth factors, and hormones regulating proliferation, apoptosis, survival, differentiation, and malignant transformation [66, 67]. Erk1/2 is a crucial signaling pathway in cell response to oxidative stress injury [68, 69]. It was also reported that activation of Erk1/2 could correct the loss of mitochondria membrane potential in a temporal correlation with protection towards apoptotic cell death [70]. Several proposals suggest that phosphorylated and activated Erk1/2 could regulate the activity of some transcription factors, which may contribute to the protective effect [71, 72]. Artemisinin increased the phosphorylation of Erk1/2 (Fig. 6a, b) and its downstream targets p90^RSK^ and CREB (Fig. 8b, c) in temporal correlation with increased level of expression of Bcl-2 (Fig. 8d). And inhibition of Erk1/2 by application of Erk1/2 pathway inhibitor PD98059 and knockdown expression of Erk1/2 or knockdown of upstream gene KRAS by siRNA significantly blocked the protective effect of artemisinin (Fig. 7). These results clearly indicated that the activation of Erk1/2 is involved in the protective effect of artemisinin in BMSCs.

The Bcl-2 protein family is classified into three subgroups with distinct structures: anti-apoptotic proteins like Bcl-2 and Bcl-xL, pro-apoptotic proteins like Bax, and Bcl-2 homology 3 domain (BH3)-only proteins such as Bad [73]. The dynamic balance between anti-apoptotic Bcl-2 (Bcl-xL) and pro-apoptotic Bax proteins play an important role in determining the fate of cells during ischemia [74]. Accumulating evidence has shown that an increase in the ratio of Bcl-2 (Bcl-xL)/Bax inhibits Bax translocation to the mitochondria and then protects cells against apoptotic insults; however, a shift in the balance towards an excess of Bax evokes ischemia-induced apoptosis [75]. Therefore, Bcl-2 played a crucial role in the protective action against cell death induced by oxidative stress by different mechanisms including the regulation of mitochondrial bioenergetics [76–79]. In the present study, we found that artemisinin upregulated the level of expression of Bcl-2, findings in line with the above concept, and proposing a mechanistic explanation to the protective effects of artemisinin to BMSCs exposed to H_2_O_2_ oxidative stress insult.

**Fig. 9:**
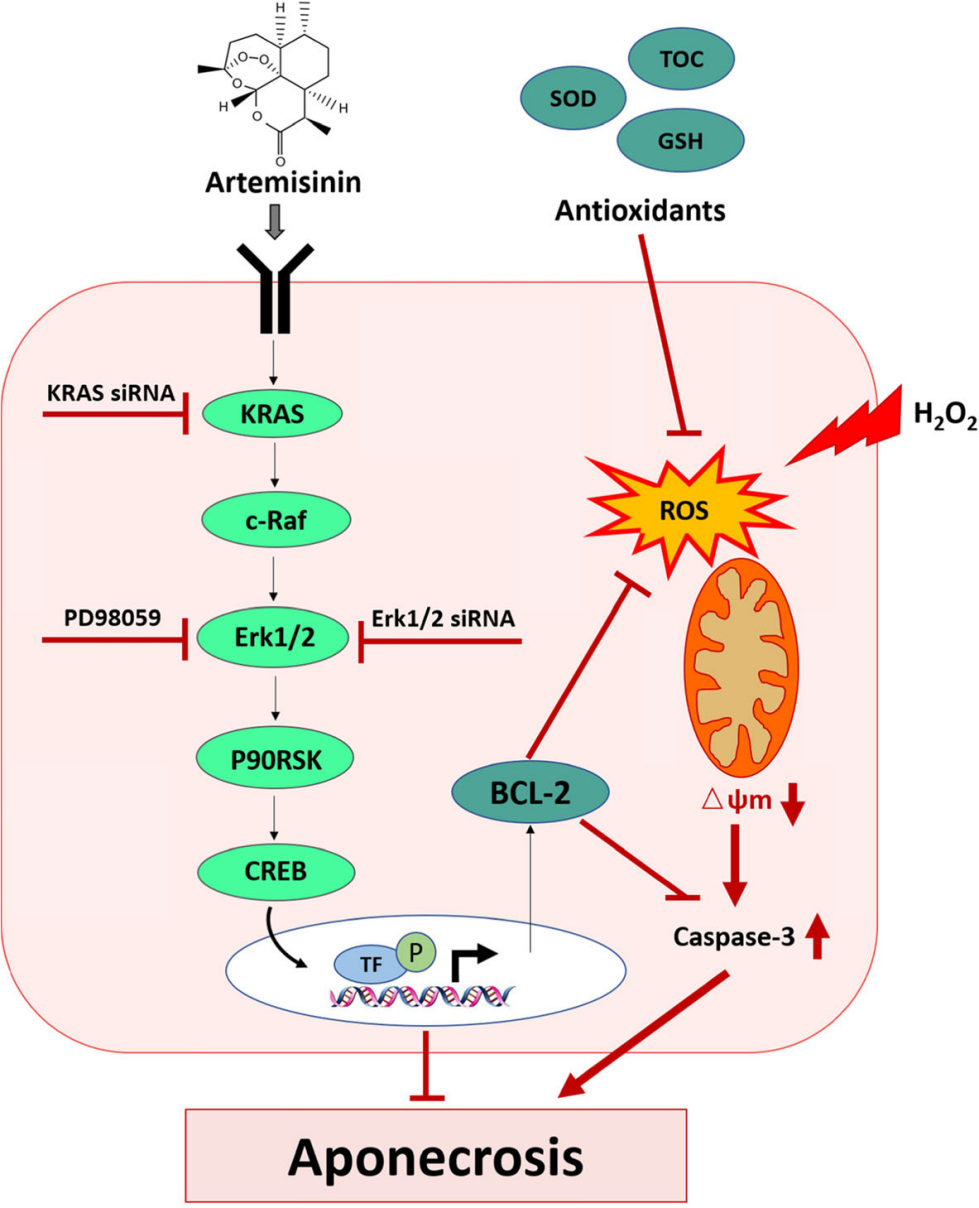
Schematic representation of convergent pathways induced by artemisinin for cytoprotection of BMSCs towards H_2_O_2_-induced aponecrotic cell death. Black arrows—physiological pathways; red arrows—pathological pathways; red bars—inhibitory effect

Both PI3K and Erk1/2 signaling pathways have been characterized as cellular mechanisms of anti-apoptotic and anti-oxidative stress defenses [80–85]. In present study, we did not observe any effect of artemisinin on the activation of PI3K; however, the phosphorylation of Erk1/2 was significantly stimulated by artemisinin pretreatment. The protective effect was blocked when the Erk1/2 signaling pathway was inhibited by PD98059 (Fig. 6c), knockdown of the expression of Erk1/2 by siMAPK1 and siMAPK3 (Fig. 7a–d) or knockdown of the upstream gene KRAS by siKRAS (Fig. 7e–h) while the inhibitor of Akt pathway LY294002 did not affect the protective effect of artemisinin on BMSCs (Fig. 6d). These findings indicate a temporal causal correlation between the protective effects of artemisinin towards H_2_O_2_-induced oxidative stress and the ability of artemisinin to stimulate the phosphorylation of Erk1/2. Consistent with these findings, artemisinin stimulated phosphorylation of the downstream substrates p90^RSK^ and CREB indicating that the whole c-Raf, Erk1/2, p90^RSK^, CREB signaling pathway was activated by artemisinin. Accumulating evidence indicates that activated Erk1/2 phosphorylates p90^RSK^ and then triggers the phosphorylation of Bad, resulting in protection towards oxidative ischemic insults [86]. During cerebral ischemia, p-p90^RSK^ phosphorylates the pro-apoptotic protein Bad and p-Bad subsequently prevents Bad interaction with Bcl-2 and inhibits pro-apoptotic protein Bax translocation to the mitochondria in the ischemic brain [87]. Erk1/2/p90^RSK^ play key roles in the activation of CREB in the ischemic tissue [88]. The transcription factor CREB plays important roles in the regulation of various cellular responses, like proliferation, and survival in a variety of cell types exposed to oxidative stress [89]. According to present results, we propose that artemisinin-induced activation of c-Raf-Erk1/2-p90^RSK^-CREB-Bcl-2-related anti-apoptotic signaling might be involved in inhibition of caspase 3 activity and other cell death reactions, thereby conferring cytoprotection (Fig. 9).

In conclusion, we demonstrated that artemisinin pretreatment of BMSCs mediates a postconditioning benefit which protects them towards H_2_O_2_ oxidative stress injury. Primary cytoprotective mechanisms involve decrease ROS, activation of c-Raf-Erk1/2-p90^RSK^-CREB, increased expression of Bcl-2, and attenuation of Ψm. The experiments presented, therefore, bridge a gap in knowledge between the transient benefits of BMSC transplantation seen in vivo and a novel therapeutic proposal of using artemisinin to achieve a more efficient protective capacity of transplanted BMSCs in ischemic tissues environment.

## Data Availability

All data generated or analyzed during this study are included in this published article.

## Abbreviations

ACTs: Artemisinin-based combination treatments
Akt: Proto-oncogene phosphoinositide 3-kinase-activated serine/threonine kinase
Bcl-2: B cell lymphoma 2 protein
BGP: Glycerol 2-phosphate
BMSCs: Bone marrow-derived mesenchymal stem cells
BSA: Bovine serum albumin
CAT: Catalase
CREB: cAMP response element binding protein
DMSO: Dimethyl sulfoxide
Erk1/2: Extracellular-signal-regulated kinase 1/2
FACS: Fluorescence-activated cell sorting
FBS: Fetal bovine serum
GPx: Glutathione peroxidase
H_2_O_2_: Hydrogen peroxide
IBMX: 3-Isobutyl-1-methylxantine
MTT: 3-(4,5-Dimethylthiazol-2-yl)-2, 5-diphenyltetrazolium bromide
NC: Nitrocellulose
OD: Optical density
p90^rsk^: p90 ribosomal s6 kinase
PI: Propidium iodide
ROS: Reactive oxygen species
SD: Standard deviation
SOD: Superoxide dismutase
